# Genetic and pharmacological inhibition of two‐pore domain potassium channel TREK‐1 alters depression‐related behaviors and neuronal plasticity in the hippocampus in mice

**DOI:** 10.1111/cns.13450

**Published:** 2020-08-30

**Authors:** Fangfang Wu, Hongbin Sun, Weigang Gong, Xiaoli Li, Zhaohui Pan, Han Shan, Zhijun Zhang

**Affiliations:** ^1^ Department of Neurology, Affiliated ZhongDa Hospital, Neuropsychiatric Institute, School of Medicine Southeast University Nanjing China; ^2^ School of Life Science and Technology Shanghai Tech University Shanghai China; ^3^ Department of Neurology, Qilu Hospital Shandong University Jinan China; ^4^ Department of Pharmacy Fudan University Shanghai Cancer Center Shanghai China

**Keywords:** depression, hippocampus, mice, neuronal plasticity, TREK‐1

## Abstract

**Introduction:**

The two‐pore domain potassium channel TREK‐1 is a member of background K^+^ channels that are thought to provide baseline regulation of membrane excitability. Recent studies have highlighted the putative role of TREK‐1 in the action of antidepressants, and its antagonists might be potentially effective antidepressants. However, the mechanisms underlying the actions of TREK‐1 are not yet fully understood.

**Methods:**

The expression of TREK‐1 was examined in a mouse model of chronic unpredictable mild stress (CUMS) using immunoblotting. Neuron‐specific genetic manipulation of TREK‐1 was performed through adeno‐associated virus. Behavioral tests were performed to evaluate depression‐related behaviors. Electrophysiological recordings were used to evaluate synaptic plasticity. Golgi staining was used to examine neuroplasticity.

**Results:**

TREK‐1 expression was increased in the mouse hippocampus after CUMS. Knockdown of TREK‐1 in hippocampal neurons significantly attenuated depressive‐like behaviors and prevented the decrease of CUMS‐induced synaptic proteins in mice. Further examination indicated that neuron‐specific knockdown of TREK‐1 in the hippocampus prevented stress‐induced impairment of glutamatergic synaptic transmission in the CA1 region. Moreover, chronic TREK‐1 inhibition protected against CUMS‐induced depressive‐like behaviors and impairment of synaptogenesis in the hippocampus.

**Conclusion:**

Our results indicate a role for TREK‐1 in the modulation of synaptic plasticity in a mouse model of depression. These findings will provide insight into the pathological mechanism of depression and further evidence for a novel target for antidepressant treatment.

## Graphical abstract

TREK‐1 channel in the hippocampus is essential for depressive‐like behaviors in response to the CUMS‐induced anhedonia model of depression. This study demonstrated the modulatory effect of TREK‐1 on dendrite morphology, dendrite spine density, glutamatergic synaptic transmission, and synaptogenesis in the hippocampus. TREK‐1 is involved in the pathogenesis of depression by regulating neuronal plasticity.

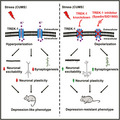

## 1 INTRODUCTION

Major depressive disorder (MDD) is one of the most common mental disorders of which almost one in five people will suffer from at some point in their lifetime and is associated with grave consequences.^1^, ^2^ Despite its considerable burden, efforts to develop novel interventions have been hindered by a limited understanding of the underlying neurobiology. Currently, pharmaceutical treatments are the most common strategy for MDD, but use of these medications is limited by slow onset, side effects, and low efficacy.^3^, ^4^ MDD is a highly stress‐sensitive illness,^5^ and the hippocampus is a highly stress‐sensitive brain region.^6^, ^7^ Basic and clinical studies revealed that depression is associated with reduced size and decreased neuronal synapses in the brain regions that regulate mood and cognition, including the hippocampus.^8^ Stress and depression cause atrophy of neurons, decreased dendrite length and branching, and spine loss in the hippocampus.^8^ Therefore, research efforts in the field are focused on identifying the neurobiological mechanisms underlying those novel antidepressant actions.^9^, ^10^

Mechano‐gated and arachidonic acid‐activated tandem of P domains in a weak inwardly rectifying K^+^ channel (TWIK)‐related K^+^ 1 (TREK‐1) is a member of the two‐pore‐domain K^+^ (K_2P_) channel family.^11^ The *TREK‐1* gene is highly expressed in the brain, especially in the hippocampus, prefrontal cortex, hypothalamus, and midbrain.^12^ It has previously been revealed that TREK‐1 displays a key role in depression.^13^ TREK‐1, which is expressed both presynaptically and postsynaptically,^14^ influences neuronal resting membrane potential, action potential duration, membrane input resistance, and neurotransmitter release.^15^ Therefore, regulating the TREK‐1 opening in presynaptic neurons is anticipated to affect neurotransmitter release.

A previous study demonstrated that TREK‐1‐deficient mice displayed a depression‐resistant phenotype by increasing the efficacy of 5‐hydroxytryptamine (5‐HT) neurotransmission and reducing the elevation of corticosterone levels under stress.^13^ Furthermore, Mazella et al reported that a sortilin‐derived peptide, named spadin, acted as a TREK‐1 channel blocker and induced a strong antidepressant effect at 4 days after injection.^16^ Moreover, it has been reported that TKDC, an inhibitor targeting TREK‐1, exhibited obvious antidepressant‐like effects.^17^ We previously reported that a screened TREK‐1 inhibitor (SID1900) induced a significant antidepressant response in a rat model of chronic unpredictable mild stress (CUMS).^18^ Moreover, the mechanism of the antidepressant action of spadin was related to the upregulation of the expression of brain‐derived neurotrophic factor and synaptic proteins.^19^ Both SID1900 and spadin substantially potentiated 5‐HT neurotransmission in the dorsal raphe nuclei and the prefrontal cortex.^16^, ^18^ Recently, Kim et al reported that TREK‐1 in the hippocampal neurons had antidepressant effects in a lipopolysaccharide‐induced acute depression model.^20^ However, no studies have demonstrated the potential effects or cellular mechanisms involved in the role of hippocampal TREK‐1 channels on depressive‐like behaviors in chronic depression model.

Therefore, in the current study, we investigated the effect of genetic and pharmacological TREK‐1 inhibition in the hippocampus on antidepressant efficacy in a mouse CUMS model using neuron‐specific genetic manipulation of TREK‐1 via adeno‐associated virus (AAV) and the TREK‐1 inhibitors spadin and SID1900. Additionally, the underlying mechanisms involved in TREK‐1 inhibition in this model of depressive behavior were explored. Thus, we assessed whether knockdown or inhibition of TREK‐1 in the hippocampus induced markers of neuronal plasticity, including synaptic transmission, synaptogenesis, and neurostructural plasticity. We hypothesized that chronic stress increased TREK‐1 expression in the hippocampus, thereby contributing to the impairment of synaptic transmission, eventually leading to impairment of long‐term potentiation and synaptogenesis, ultimately resulting in depressive‐like behaviors.

## 2 METHODS AND MATERIALS

### 2.1 Animals

Adult male C57BL/6J mice (20.0‐25.0 g, 5‐8 weeks old) were purchased from the Model Animal Research Center of Nanjing University (Nanjing, China) and randomly assigned to experimental groups. Animals were housed under constant temperature and humidity and a 12‐h light/12‐h dark cycle. Food and water were available ad libitum. Animal procedures were performed in strict accordance with the Animal Research: Reporting of In Vivo Experiments guidelines. The care and use of animals were reviewed and approved by the Institutional Animal Care and Use Committee at the Medical School of Southeast University.

### 2.2 CUMS procedure

To induce chronic stress in mice, we used a previously validated CUMS procedure with slight modifications.^21^ Briefly, mice were individually housed and subjected to various, randomly scheduled stressors, which were mild and unpredictable in nature, duration, and frequency 2‐3 times a day for 5 weeks. Stressors included food deprivation, water deprivation, inversion of day/night light cycle, absence of sawdust in the cage, soiled cage bedding, tail nipping, restraint, 45° tilted cage, and pairing with another stressed animal. Multiple behavioral tests were performed on the same animals. Animals from different groups underwent the same tests in the same order. Sucrose preference and body weight of each animal were evaluated weekly until the end of the CUMS.

### 2.3 Construction, preparation, and infusion of recombinant adeno‐associated virus

Adeno‐associated virus expressing a short hairpin RNA (shRNA) targeting the sequence of the TREK‐1 gene (AAV‐shRNA‐TREK‐1), AAV overexpressing TREK‐1 (AAV‐TREK‐1), and a negative control AAV (AAV‐Con) were purchased from OBiO Technology Co., Ltd. All viral particles express mCherry under the synapsin‐1 (SYN) promoter (a neuron‐specific promoter) flanked by the woodchuck hepatitis posttranscriptional regulatory element. For intra‐hippocampal microinjections of neuron‐specific AAV, a total volume of 2.0 μL viral preparations (2 × 10^10^ viral genomes in a volume of 1.0 μL per side, Obio) was delivered bilaterally into the hippocampal CA1 and dentate gyrus (DG) regions (AP = −2.0 mm, ML = ±1.5 mm, DV [CA1] = −1.5 mm or DV [DG] = −2.0 mm, relative to Bregma],^22^ and the virus solution (0.5 μL virus solution each point) was infused at a rate of 0.1 μL/min, followed by 10 min of rest to allow diffusion. For a full description of recombinant AAV 2/8 serotype virus preparation and stereotaxic microinjection surgeries, please refer to the [Link], [Link].

To evaluate the effect of TREK‐1 shRNA or overexpression in the CUMS model, mice were randomly divided into groups at 4 weeks after microinjection of AAV as follows: control + AAV‐Con; control + AAV‐shRNA‐TREK‐1; CUMS + AAV‐Con; CUMS + AAV‐shRNA‐TREK‐1; or control + AAV‐Con; control + AAV‐TREK‐1; CUMS + AAV‐Con; CUMS + AAV‐TREK‐1. Subsequently, mice were exposed to a CUMS or control protocol procedure for 5 weeks.

### 2.4 Drugs and drug treatments

TREK‐1 channel inhibitor (N‐[2‐[(1S, 4S, 5S)‐5‐bicyclo [2.2.1] hept‐2‐ enyl] ethyl]‐5‐[(2, 4‐ difluorophen‐oxy) methyl]‐1, 2‐oxazole‐3‐carboxamide, SID1900) was provided by the National Chemical Library in the Shanghai Institute of Materia Medica (SIMM) affiliated Chinese Academy Sciences (Shanghai, China) as previously described.^18^ Spadin (10^−5^ mol/L in a 100 µL bolus) (Shanghai Mocell Biotech Co. Ltd.), SID1900 (7 mg/kg), or the vehicle (saline) were administered in an intraperitoneal injection (i.p.) fashion.^18^, ^23^ All solutions were prepared as previously described,^18^ with some dose equivalent conversion.

After 5 weeks of stress procedure, body weight was evaluated, and the sucrose preference test (SPT) and forced swimming test (FST) were performed for all mice. Only stressed mice that exhibited decreased sucrose preference and increased immobility in FST (39 in 56 mice, succumbed rate 69.6%) were enrolled in the following drug‐treatment (i.p.) experiments, whereas the remaining mice (17 in 56 mice) were excluded from the experiment. Mice were randomly divided into the following groups: control + saline; control + spadin; control + SID1900; CUMS + saline; CUMS + spadin; CUMS + SID1900.

### 2.5 Behavioral tests

Mice were habituated in the procedure room for at least 3 hours before testing was performed. All tests were carried out during the dark cycle (7:00 am‐7:00 pm) in a sound‐attenuated room under low‐intensity light and were scored by the same person. Behavior was monitored through a video camera that was positioned in front of the testing apparatus, and the images were analyzed using ANY‐maze behavioral analysis software (version 4.3; Stoelting Co.) and ForcedSwimScan™ (Clever Sys Inc) by an experienced investigator who was blind to the treatment regimen of the animals tested. For a detailed description of the SPT, open‐field test (OFT), and FST, please refer to the [Link], [Link].

### 2.6 Electrophysiological recordings

Electrophysiological recordings of synaptic plasticity were performed in 300‐μm hippocampal slices, as described previously.^24^ Briefly, a bipolar electrode was placed in the Schaffer collaterals and evoked field excitatory postsynaptic potentials (fEPSP) were recorded with a glass micropipette (3 mol/L NaCl; 3‐5 MΩ resistance) that was placed in the stratum radiatum layer of the CA1 region. Long‐term potentiation (LTP) was induced with high‐frequency stimulation (HFS) train (100 Hz for 1 second) and was quantified as the percentage change between the fEPSP slopes 60 minutes after and 20 minutes before the train. Whole‐cell voltage‐clamp recordings of CA1 pyramidal cells were recorded using patch electrodes (3‐6 MΩ resistance). Additionally, miniature excitatory postsynaptic current (mEPSC) was isolated by including bicuculline (20 μmol/L) and tetrodotoxin (1 μmol/L) in the bath solution. For complete technical details of slice electrophysiology and analysis, please refer to the [Link], [Link].

### 2.7 Golgi‐Cox staining, imaging, and analysis

After the CUMS procedure and final behavior tests, animals were sacrificed. Freshly dissected mouse brains were used for Golgi‐Cox staining with the FD Rapid GolgiStain kit (FD Neuro Technologies) according to the manufacturer's protocol. Briefly, mouse brains were incubated in Golgi solution A + B for 10 days and then incubated for 3 days in solution C. Coronal sections (120 μm) were cut using a cryostat (CM1950, Leica) and stained. Images of Golgi‐stained neurons were acquired on an OLYMPUS microscope (OLYMPUS, Tokyo, Japan, DP73) using the Optical Fractionator method with Microbrightfield Stereo Investigator software (Stereo Investigator software; Microbrightfield). From optical cell images, dendrites were traced using NeuronJ software (ImageJ Plugin, www.imagescience.org/meijering/software/neuronj, version 1.46r; NIH). The total length of dendrites was calculated as the sum of the dendritic length from one neuron. Sholl analysis was used to assess the complexity of neural dendrites by placing concentric circles in 10‐μm increments starting at 10 μm from the soma. The number of dendritic intersections with each ring was counted, and the results are reported as the number of intersections per radial distance from the soma and the total number of intersections. Per condition, 30 neurons from 4 animals were used for calculation of dendrite length and Sholl analysis in a blinded manner. To calculate spine density, we traced and measured a length of dendrite and counted the number of spines along the dendrite. Spine density was calculated from the number of spines divided by the dendrite length. Per group, 40 neurons from 4 animals were analyzed in a blinded manner for spine density analysis.

### 2.8 Western blot analysis and other experiments

Western blot analysis was performed as previously described.^21^ Microscopy and image analysis were performed as described in the [Link], [Link].

### 2.9 Statistical analysis

Statistical analysis was performed using GraphPad Prism 6 Software (GraphPad Software Inc). Data were presented as the mean ± standard error of the mean (SEM). Statistical analyses used for the different experiments are described in respective figure legends. Significance was assessed by Student's *t*‐tests (two‐tailed) for comparisons of two groups or Mann‐Whitney *U*‐test for non‐normally distributed data. Brown‐Forsythe test was first used to evaluate the homogeneity of variance. Two‐way ANOVA followed by Tukey's multiple comparison test was employed for 4 or more groups. Behavioral data collected at multiple, sequential time points (ie, for a single animal at 9 time points) were analyzed using two‐way repeated‐measures ANOVA, followed by Tukey's multiple comparison test. *P* < .05 was considered as statistically significant.

## 3 RESULTS

### 3.1 Neuron‐specific knockdown of TREK‐1 in the mouse hippocampus prevents chronic stress‐induced depressive‐like behaviors

**FIGURE 1:**
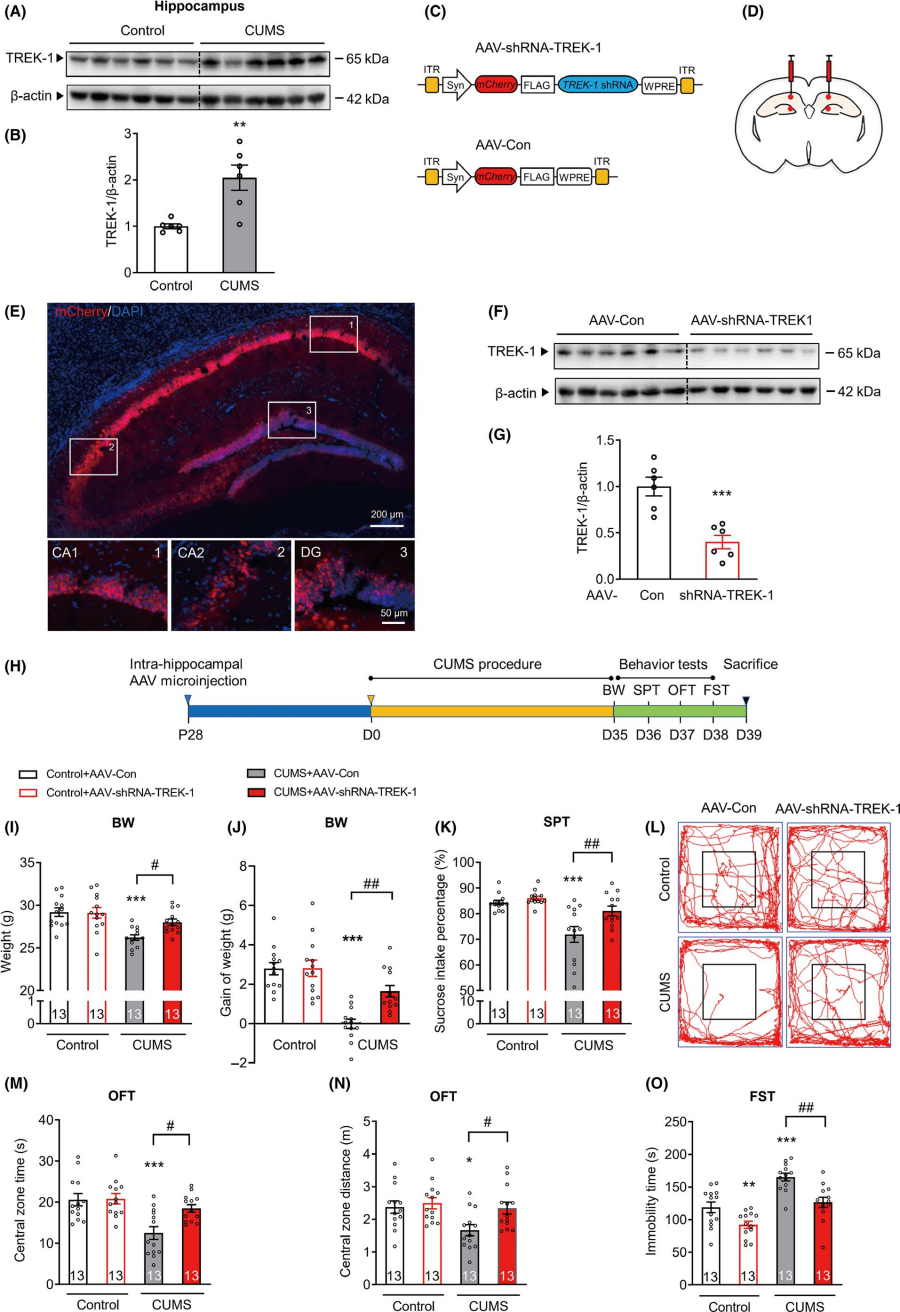
Neuron‐specific knockdown of TREK‐1 in the mouse hippocampus prevents chronic stress‐induced depressive‐like behaviors. A and B, Images (A) and a bar graph (B) showing Western blot analysis of TREK‐1 expression in the hippocampus of CUMS mice. After exposure to CUMS for 5 wk and finished behavior tests, mice were sacrificed. n = 6 animals per group. **P < .01 versus control using Student's *t*‐test. C, Schematics of the AAV constructs (AAV2/8 serotype) for neuron‐specific knockdown of TREK‐1 (AAV‐shRNA‐TREK‐1) or negative control (AAV‐Con) used in the experiments presented in D‐O. D, Illustration of bilateral viral injections into the mouse hippocampus. E, Representative fluorescence images of the mouse hippocampus after AAV infection. Blue represents nuclei stained with DAPI. Red represents mCherry‐TREK‐1‐shRNA‐AAV. Scale bar, 200 or 50 μm. F and G, The expression of TREK‐1 in the hippocampus at 4 wk after the TREK‐1‐shRNA‐AAV microinjection was determined by Western blot analysis. n = 6 animals per group. ***P < .001 versus AAV‐Con control using Student's *t*‐test. H, Illustration of the experimental procedure shown in I‐O. Four weeks after Con or shRNA‐TREK‐1 AAV microinjection in the hippocampus, mice were exposed to a CUMS or control protocol. Body weight, sucrose preference test, open‐field test, and forced swim test were measured after 5 wk of CUMS exposure. I‐J, Body weight (I) and body weight gain (J) at 5 wk after CUMS. n = 13 animals per group. ***P < .001 versus control AAV‐Con group; #*P* < .05 and ##*P* < .01 versus CUMS AAV‐Con group using two‐way ANOVA followed by Tukey's test. K, Sucrose preference at 5 wk after CUMS. n = 13 animals per group. ***P < .001 versus control AAV‐Con group; ##*P* < .01 versus CUMS AAV‐Con group using two‐way ANOVA followed by Tukey's test. L‐N, Representative moving paths (L) and bar graphs (M‐N) of control and CUMS mice in the open‐field test. After 5 wk of CUMS exposure, AAV‐shRNA‐TREK‐1‐injected mice displayed a significant increase in the central zone time (M) and central zone distance (N) of the open field when compared with their respective AAV‐Con in the open‐field test. Black lines represent the size of the center zone provided for the OFT. n = 13 animals per group. *P < .05 and ***P < .001 versus control AAV‐Con group; #*P* < .05 versus CUMS AAV‐Con group using two‐way ANOVA followed by Tukey's test. O, Forced swim test at 5 wk after CUMS. n = 13 animals per group. **P < .01 and ***P < .001 versus control AAV‐Con group; ##*P* < .01 versus CUMS AAV‐Con group using two‐way ANOVA followed by Tukey's test. CUMS, chronic unpredictable mild stress. ITR, inverted terminal repeats. Syn, synapsin I promoter. WPRE, woodchuck hepatitis virus posttranscriptional regulatory element. BW, body weight. SPT, sucrose preference test. OFT, open‐field test. FST, forced swim test. Numbers in bars, numbers of mice. Data are shown as mean ± SEM

In this study, we investigated the expression of TREK‐1 in the hippocampus of CUMS mice or controls by Western blot analysis. Our data showed that TREK‐1 expression increased in the hippocampus of CUMS mice (Figure 1A,B and Figure S1). To validate the role of TREK‐1 in the pathogenesis of depression in vivo, neuron‐specific AAV with a synapsin‐1 (SYN) promoter shRNA was employed to inhibit TREK‐1 expression in neurons by microinjection into the bilateral hippocampus (Figure 1C,D, Figure S2, and Table S1). mCherry was widely expressed in the hippocampus 28 d after injection (Figure 1E). Moreover, decreased TREK‐1 expression was observed in AAV‐shRNA‐TREK‐1‐injected mice when compared with AAV‐Con‐injected mice (Figure 1F,G). Four weeks after AAV microinjection, mice were exposed to a CUMS protocol for 5 weeks; then, depressive‐like behavioral tests were performed (Figure 1H). Our data showed that CUMS treatment decreased body weight and sucrose preference when compared with that of mice in the control group (AAV‐Con). This deficit was significantly ameliorated by AAV‐shRNA‐TREK‐1 (Figure 1I‐K). As shown in Figure 1L‐N and Figure S3, AAV‐shRNA‐TREK‐1 attenuated the decrease of both time and travel distance in the central zone of the open field after undergoing the CUMS paradigm. In FST, AAV‐shRNA‐TREK‐1 mice exhibited a significant decrease in the duration of immobility following 5 weeks of CUMS (Figure 1O). Together, these data demonstrate that specific knockdown of TREK‐1 in hippocampal neurons reduces depressive‐like behaviors in adult mice.

### 3.2 Neuron‐specific overexpression of TREK‐1 in the mouse hippocampus aggravates chronic stress‐induced depressive‐like behaviors

**FIGURE 2:**
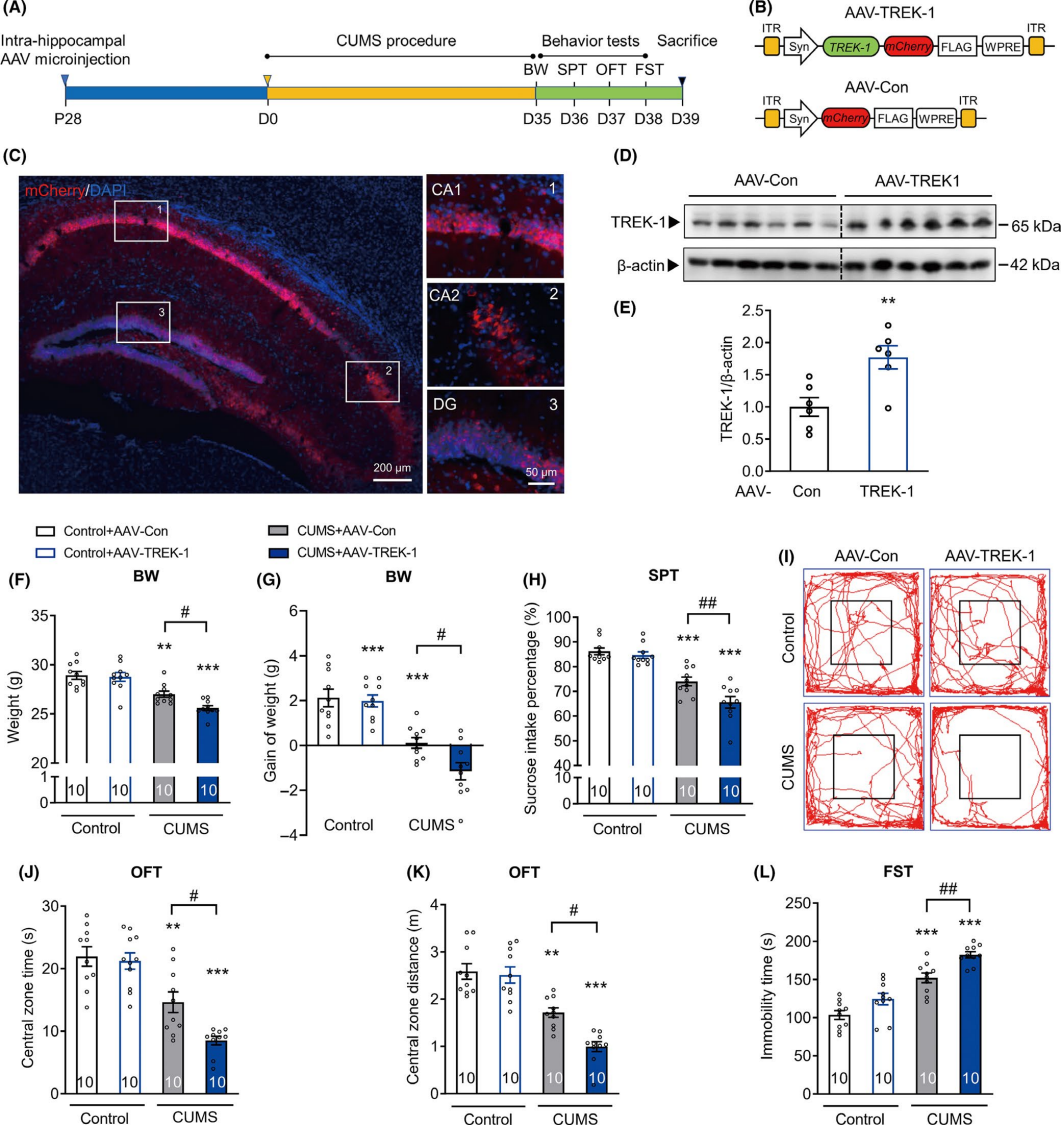
Neuron‐specific overexpression of TREK‐1 in the mouse hippocampus aggravates chronic stress‐induced depressive‐like behaviors. A, Illustration of the experimental procedure shown in B‐L. Four weeks after AAV‐Con or AAV‐TREK‐1 microinjection in the hippocampus, mice were exposed to a CUMS or control protocol. Body weight, sucrose preference test, open‐field test, and forced swim test were measured after 5 wk of CUMS exposure. B, Structure of the AAV virus (AAV2/8 serotype) for neuron‐specific overexpression of TREK‐1 (AAV‐TREK‐1) or control (AAV‐Con) used in the experiments presented in C‐L. C, Representative fluorescence images of the mouse hippocampus after AAV infection. Blue represents nuclei stained with DAPI. Red represents TREK‐1 co‐expressed with mCherry. Scale bar, 200 or 50 μm. D and E, The expression of TREK‐1 in the hippocampus at 4 wk after the AAV‐ TREK‐1 microinjection was determined by Western blot analysis. n = 6 animals per group. **P < .01 versus AAV‐Con control using Student's *t*‐test. F and G, Body weight (F) and body weight gain (G) at 5 wk after CUMS. n = 10 animals per group. **P < .01 and ***P < .001 versus control AAV‐Con group; #*P* < .05 versus CUMS AAV‐Con group using two‐way ANOVA followed by Tukey's test. H, Sucrose preference at 5 wk after CUMS. n = 10 animals per group. ***P < .001 versus control AAV‐Con group; ##*P* < .01 versus CUMS AAV‐Con group using two‐way ANOVA followed by Tukey's test. I‐K, Representative moving paths (I) and bar graphs (J‐K) of control and CUMS mice in the open‐field test. After 5 wk of CUMS exposure, AAV‐TREK‐1 microinjection in the hippocampus significantly decreased the central zone time (J) and central zone distance (K) in the open‐field test. Black lines represent the size of the center zone provided for the OFT. n = 10 animals per group. **P < .01 and ***P < .001 versus control AAV‐Con group; #*P* < .05 versus CUMS AAV‐Con group using two‐way ANOVA followed by Tukey's test. L, Forced swim test at 5 wk after CUMS. n = 10 animals per group. ***P < .001 versus control AAV‐Con group; ##*P* < .01 versus CUMS AAV‐Con group using two‐way ANOVA followed by Tukey's test. CUMS, chronic unpredictable mild stress. BW, body weight. SPT, sucrose preference test. OFT, open‐field test. FST, forced swim test. ITR, inverted terminal repeats. Syn, synapsin I promoter. WPRE, woodchuck hepatitis virus posttranscriptional regulatory element. Numbers in bars, numbers of mice. Data are shown as mean ± SEM

Next, we sought to validate the effect of neuron‐specific overexpression of TREK‐1 in the hippocampus on CUMS‐induced depressive‐like behaviors (Figure 2A). In brief, AAV with the SYN promoter vector encoding TREK‐1 (AAV‐TREK‐1) was injected into the bilateral hippocampus (Figure 2B). As expected, mCherry was widely expressed in the hippocampus 28 d after injection (Figure 2C). Increased expression of TREK‐1 was observed in AAV‐TREK‐1‐injected mice when compared to AAV‐Con‐injected mice (Figure 2D‐E). As shown in Figure 2F‐H, AAV‐TREK‐1 mice exhibited a significant decrease in body weight and sucrose intake preference when compared to AAV‐Con mice after being exposed to the CUMS paradigm. Although the CUMS procedure significantly reduced both time and distance in the open‐field central zone, the effect was significantly enhanced in AAV‐TREK‐1 mice when compared to AAV‐Con mice (Figure 2I‐K and Figure S4). Furthermore, TREK‐1 overexpression in hippocampal neurons led to a significant increase in immobility time in the FST (Figure 2L). Combined, these results demonstrated that specific overexpression of TREK‐1 in the hippocampal neurons aggravated CUMS‐induced depressive‐like behaviors.

### 3.3 Neuron‐specific knockdown of TREK‐1 blocks chronic stress‐induced synaptic plasticity impairment in the mouse hippocampus

**FIGURE 3:**
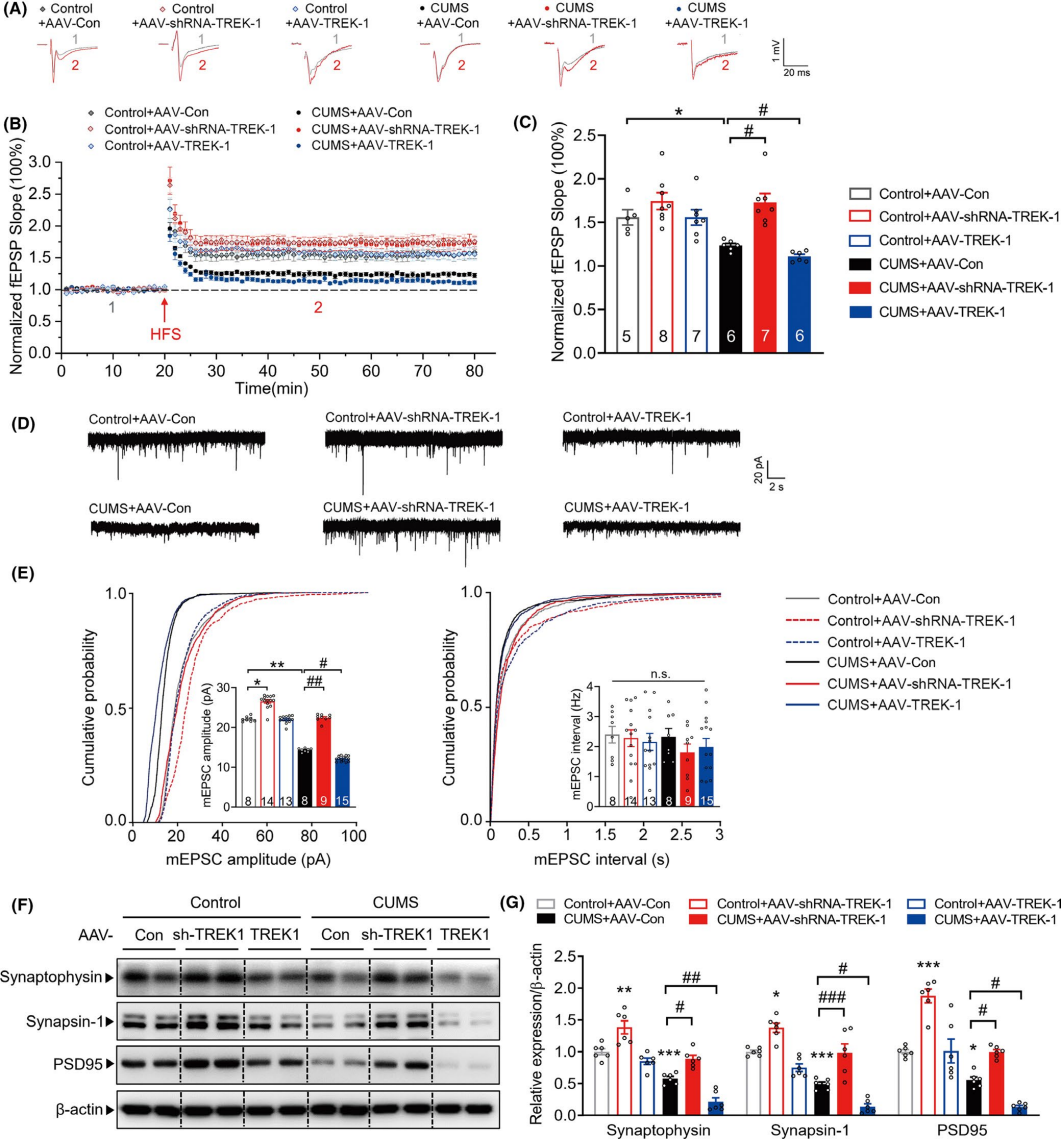
Neuron‐specific knockdown of TREK‐1 blocks chronic stress‐induced synaptic plasticity impairment in the mouse hippocampus. A‐C, Representative traces (A), summary plots (B), and averaged fEPSP slope (C) of LTP induced by HFS in acute slices from different groups at 5 wk after CUMS. Sample traces (A) were taken at time points 1 and 2 indicated in the summary plots (B). C, Histogram showing the LTP magnitude averaged from the last 15 min of recordings from different groups. n = 5‐8 slices from four animals per group. *P < .05 versus control AAV‐Con group; #*P* < .05 versus CUMS AAV‐Con group using two‐way ANOVA followed by Tukey's test. D‐E, Representative traces (D) and quantification of amplitude (E, left) and frequency (E, right) of mEPSCs in hippocampal CA1 pyramidal neurons from different groups at 5 wk after CUMS. n = 8‐15 neurons from four animals per group. *P < .05 and **P < .01 versus control AAV‐Con group; #*P* < .05 and ##*P* < .01 versus CUMS AAV‐Con group using two‐way ANOVA followed by Tukey's test. F‐G, Representative images (F) and bar graph (G) showing that AAV‐shRNA‐TREK‐1 attenuated the CUMS‐induced decrease in synaptophysin, synapsin‐1, and PSD95 expressions in the hippocampus, and overexpression TREK‐1 aggravated the decrease of synaptic proteins at 5 wk after CUMS. n = 6 animals per group. *P < .05, **P < .01 and ***P < .001 versus control AAV‐Con group; #*P* < .05, ##*P* < .01 and ###*P* < .001 versus CUMS AAV‐Con group using two‐way ANOVA followed by Tukey's test. CUMS, chronic unpredictable mild stress. fEPSP, field excitatory postsynaptic potential. HFS, high‐frequency stimulation. LTP, long‐term potentiation. mEPSC, miniature excitatory postsynaptic current. sh‐TREK‐1, shRNA‐TREK‐1. Numbers in bars, numbers of slices (C), neurons (E). Data are shown as mean ± SEM

Chronic stress results in impairment of excitatory synapses and dysregulation of glutamate signaling in rodent brains^25^, ^26^; therefore, we further examined the effect of TREK‐1 on glutamate neurotransmission in the CA1 area of the hippocampus. To test whether TREK‐1 modulates long‐term synaptic plasticity, we recorded LTP induced by HFS in the CA1 region of hippocampal slices. We first compared fEPSPs in the Schaffer collateral‐CA1 area in acute slices from control mice and mice with a CUMS‐induced depressive‐like phenotype. CUMS exposure induced suppression of LTP in the CA1 region, and this effect was normalized by AAV‐shRNA‐TREK‐1 treatment (Figure 3A‐C). One hour after HFS, neuron‐specific knockdown of TREK‐1 in the hippocampus significantly increased the slope of fEPSP, and AAV‐shRNA‐TREK‐1 slices showed normal levels of fEPSP after CUMS (Figure 3C). Moreover, compared to those of mice in the CUMS + AAV‐Con group, the fEPSP slope in mice in the CUMS + AAV‐TREK‐1 group was reduced; however, no significant differences were observed between mice in the control AAV‐Con and AAV‐TREK‐1 groups (Figure 3C). We further used whole‐cell patch clamp to assess the effect of TREK‐1 on the synaptic properties of hippocampal CA1 pyramidal neurons. As shown in Figure 3D,E, chronic stress markedly reduced the amplitude of mEPSC in the CA1 region, and AAV‐shRNA‐TREK‐1 significantly attenuated this effect. In TREK‐1‐overexpressed CA1 neurons, mEPSCs were substantially decreased in amplitude after CUMS, compared to CUMS + AAV‐Con neurons (Figure 3E). No differences were observed in mEPSC frequency among all groups.

Consistent with the impairment of synaptic plasticity, CUMS significantly decreased the expression of presynaptic protein synaptophysin, synapsin‐1, and postsynaptic protein PSD95 in the hippocampus. These effects were significantly attenuated by microinjection of AAV‐shRNA‐TREK‐1 and enhanced by microinjection with AAV‐TREK‐1 (Figure 3F,G).

### 3.4 TREK‐1 inhibitor ameliorates CUMS‐induced depressive‐like behaviors

**FIGURE 4:**
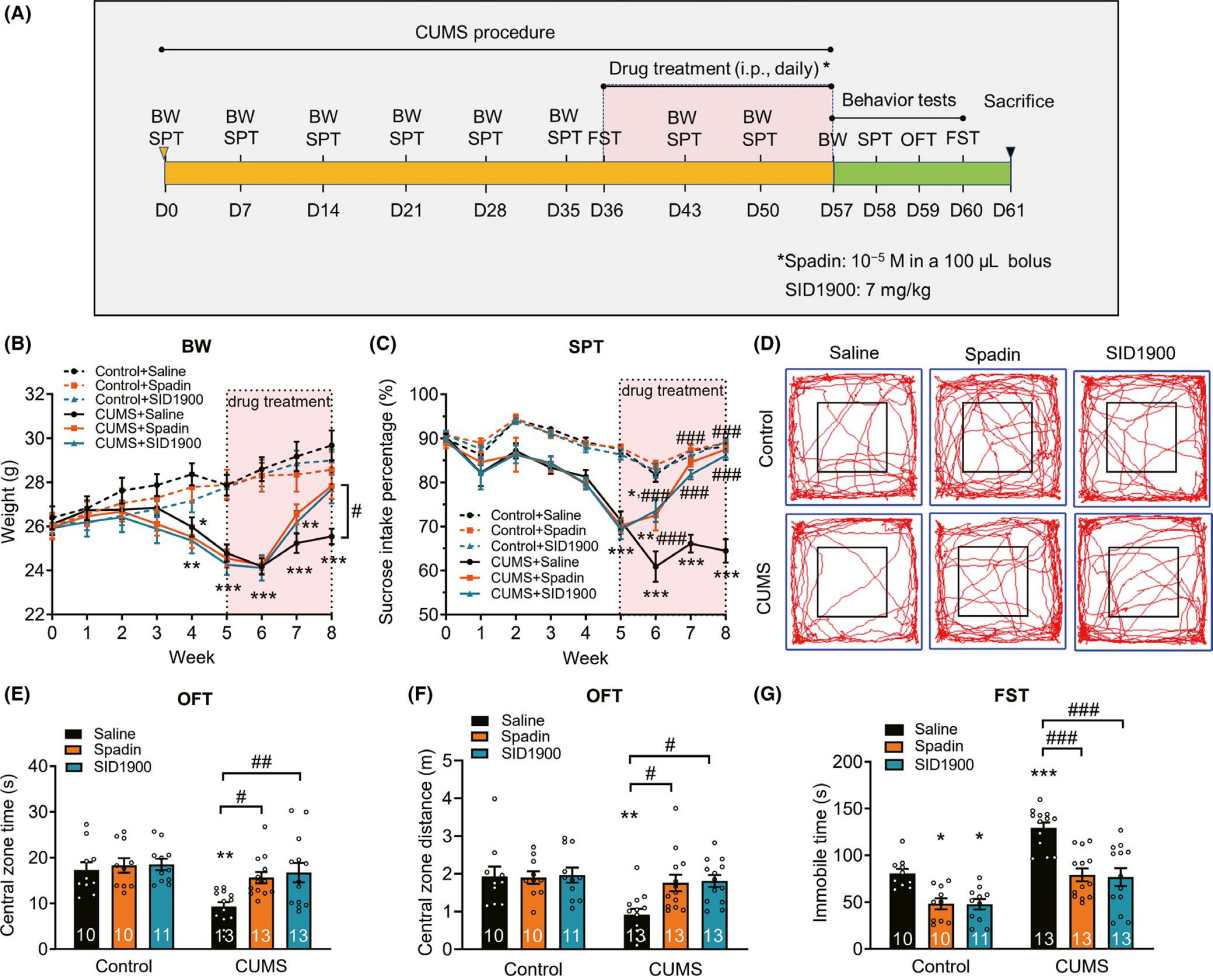
TREK‐1 inhibitor ameliorates CUMS‐induced depressive‐like behaviors. A, Illustration of the experimental procedure shown in B‐G. B and C, Body weight recording (B) and sucrose preference test (C) were performed during the experimental period to assess the depression model. n = 10, 10, 11, 13, 13, and 13 animals in the control + saline, control + spadin, control + SID1900, CUMS + saline, CUMS + spadin, and CUMS + SID1900 groups, respectively. *P < .05, **P < .01 and ***P < .001 versus control + saline group; #*P* < .05 and ###*P* < .001 versus CUMS + saline group using two‐way repeated‐measures ANOVA followed by Tukey's test. D‐F, Effect of TREK‐1 inhibitors on the moving paths (D), central zone time (E) and central zone distance (F) in OFT after CUMS exposure. Three weeks after treatment with TREK‐1 inhibitors spadin and SID1900, mice were tested in the open field. n = 10, 10, 11, 13, 13, and 13 animals in the control + saline, control + spadin, control + SID1900, CUMS + saline, CUMS + spadin, and CUMS + SID1900 groups, respectively. **P < .01 versus control + saline group; #*P* < .05 and ##*P* < .01 versus CUMS + saline group using two‐way ANOVA followed by Tukey's test. G, Effect of TREK‐1 inhibitors on the despair behaviors after CUMS exposure. Forced swim test (G) were performed at the third week of drug treatment after CUMS. n = 10, 10, 11, 13, 13, and 13 animals in the control + saline, control + spadin, control + SID1900, CUMS + saline, CUMS + spadin, and CUMS + SID1900 groups, respectively. *P < .05 and ***P < .001 versus control + saline group; ###*P* < .001 versus CUMS + saline group using two‐way ANOVA followed by Tukey's test. CUMS, chronic unpredictable mild stress. BW, body weight. SPT, sucrose preference test. OFT, open‐field test. FST, forced swim test. Numbers in bars, numbers of mice. Data are shown as mean ± SEM

Next, we examined the effect of the TREK‐1 inhibitor (spadin and SID1900) on depressive‐like behaviors (Figure 4A). Body weight and sucrose intake preference were reduced in CUMS‐exposed animals. After the third week of treatment, the decrease in body weight was reversed by treatment with spadin and SID1900 (Figure 4B). After the first week of treatment, sucrose preference was increased by spadin and SID1900, and was reversed after the second week of treatment (Figure 4C). As shown in Figure 4D‐F and Figure S5, the OFT showed a significant decrease in both the time and distance in the central zone in stressed animals. Chronic administration of spadin and SID1900 reversed this depressive‐like behavior. In FST, exposure to the CUMS procedure significantly increased the duration of immobility, and this effect was significantly reversed by treatment with spadin and SID1900 (Figure 4G). Thus, these data demonstrated that TREK‐1 inhibitor ameliorates CUMS‐induced depressive‐like behaviors.

### 3.5 TREK‐1 inhibitor prevents CUMS‐induced synaptic plasticity impairment

**FIGURE 5:**
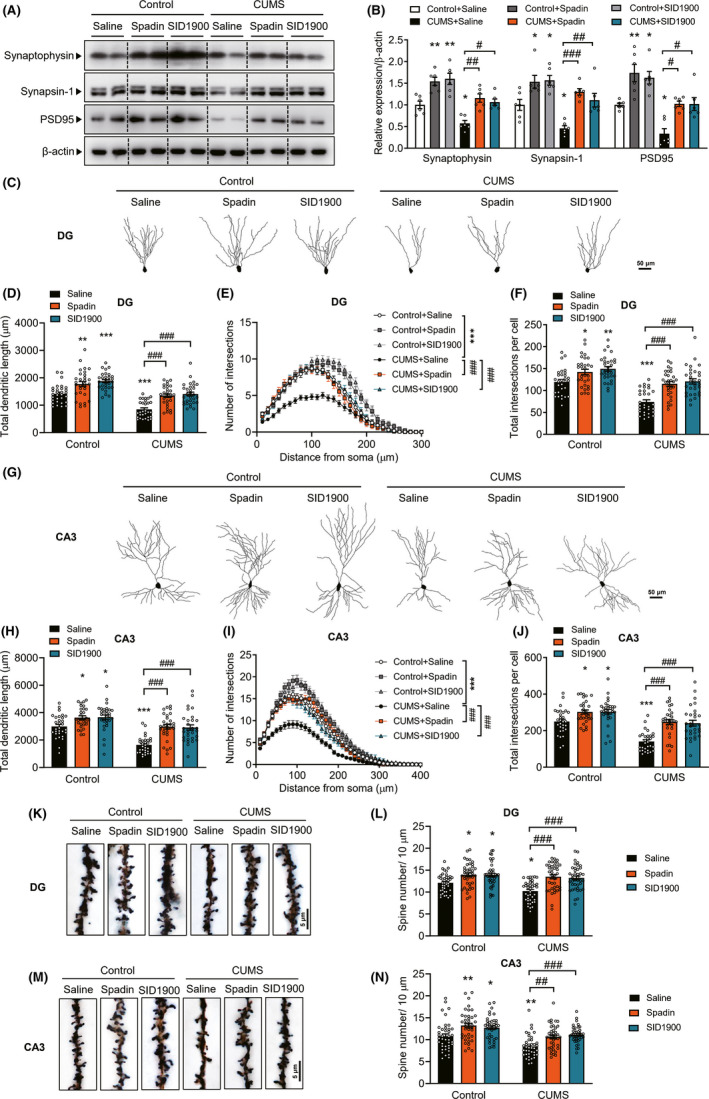
TREK‐1 inhibitor prevents CUMS‐induced synaptic plasticity impairment. A and B, Effects of TREK‐1 inhibitors on synaptic protein levels after CUMS exposure. Representative image (A) and bar graph (B) of synaptophysin, synapsin‐1, and PSD95 expression by Western blot analysis. n = 6 animals per group. *P < .05 and **P < .01 versus control + saline group; #*P* < .05, ##*P* < .01 and ###*P* < .001 versus CUMS + saline group using two‐way ANOVA followed by Tukey's test. C‐F, Morphometric analysis of Golgi‐impregnated neurons using computer‐assisted reconstructions of hippocampal dentate granule neurons. C, Representative dentate granule neurons from different groups are shown. D, Total dendritic length of dentate granule neurons in the subgranular zone from different groups are shown. E, Sholl analysis of the dendritic complexity of Golgi‐stained neurons in the dentate granule from different groups. Significant differences were observed at 40‐140 μm from the soma. The average total numbers of intersections per cell are shown in F. Thirty neurons from 4 animals per group were analyzed. Scale bar, 50 μm. *P < .05, **P < .01 and ***P < .001 versus control + saline group; ###*P* < .001 versus CUMS + saline group using two‐way ANOVA followed by Tukey's test. G‐J, Morphometric analysis of Golgi‐impregnated neurons using computer‐assisted reconstructions of hippocampal CA3 neurons. G, Representative CA3 pyramidal neurons from different groups are shown. H, Total dendritic length of CA3 pyramidal neurons from different groups are shown. I, Sholl analysis of the dendritic complexity of Golgi‐stained neurons in the CA3 from different groups. Significant differences were observed at 30‐170 μm from the soma. The average total numbers of intersections per cell are shown in J. Thirty neurons from 4 animals per group were analyzed. Scale bar, 50 μm. *P < .05 and ***P < .001 versus control + saline group; ###*P* < .001 versus CUMS + saline group using two‐way ANOVA followed by Tukey's test. K‐L, Representative image of spines (K) and bar graph of spine density (L) in the dendrites of dentate granule neurons in the subgranular zone. Forty neurons from four animals per group were analyzed. Scale bar, 5 μm. *P < .05 versus control + saline group; ###*P* < .001 versus CUMS + saline group using two‐way ANOVA followed by Tukey's test. M‐N, Representative image of spines (M) and bar graph (N) of spine density in the dendrites of CA3 pyramidal neurons. Forty neurons from four animals per group were analyzed. Scale bar, 5 μm. *P < .05 and **P < .01 versus control + saline group; ##*P* < .01 and ###*P* < .001 versus CUMS + saline group using two‐way ANOVA followed by Tukey's test. CUMS, chronic unpredictable mild stress. DG, dentate granule. Data are shown as mean ± SEM

Since TREK‐1 inhibitors ameliorated the CUMS‐induced depressive‐like phenotype, we next investigated the mechanisms underlying this process. Mice that underwent the CUMS procedure and subsequent spadin or SID1900 treatment were evaluated for synaptic plasticity impairment as determined by synaptophysin, synapsin‐1, and PSD95. As shown in Figure 5A,B, treatment with spadin and SID1900 normalized the CUMS‐induced reduction in synaptophysin, synapsin‐1, and PSD95 in the hippocampus. Besides, morphometric analysis of Golgi‐impregnated neurons revealed that CUMS significantly decreased dendritic complexity of granule cell (Figure 5C‐F) and CA3 pyramidal neurons (Figure 5G‐J), which was indicated by dendritic lengths and the number of neuronal intersections. Treatment with either spadin or SID1900 reversed this effect in both regions (Figure 5C‐J). The CUMS procedure induced a significant loss of spines in dendrites of granule cells and CA3 pyramidal neurons, and this effect was attenuated by treatment with spadin and SID1900 (Figure 5K‐N).

## 4 DISCUSSION

The data obtained in the current study shows that neuron‐specific knockdown of TREK‐1 in the hippocampus had antidepressant‐like effects in mice that underwent the CUMS procedure. In contrast, neuron‐specific overexpression of TREK‐1 in the hippocampus aggravated depressive‐like behaviors after chronic stress. Furthermore, our data showed that neuron‐specific knockdown of TREK‐1 in the hippocampus prevented chronic stress‐induced impairment of glutamate neurotransmission and synaptogenesis. Specifically, we demonstrated that chronic administration of a TREK‐1 inhibitor (spadin or SID1900) protected against CUMS‐induced depressive‐like behaviors and impairment of synaptogenesis in the hippocampus. Together, these data indicated that neuronal TREK‐1 channels regulate a depressive phenotype and synaptic plasticity after chronic stress.

Thus, we have shown that neuronal TREK‐1 channels regulate a chronic stress‐induced depressive phenotype. Neuron‐specific knockdown of TREK‐1 in the hippocampus specifically decreased depressive‐like behavior measured by the SPT, FST, and OFT in mice that received the CUMS procedure, while neuron‐specific overexpression of TREK‐1 in the hippocampus resulted in aggravated depressive‐like behaviors. This may be due to the role of neuronal TREK‐1 channels in the susceptibility of mice to a stress‐induced phenotype. TREK‐1 overexpression in hippocampal neurons resulted in increased susceptibility to chronic stress. Thus, these data suggested a direct indication of the antidepressant‐like phenotype shown by TREK‐1 knockdown and inhibition in mice after chronic stress.

Our study further revealed a detailed mechanism underlying the function of TREK‐1 in regulating the depressive phenotype after stress. Among the putative mechanisms for depression, synaptogenesis is a process that has been widely studied.^8^ TREK‐1 is highly expressed in hippocampal glutamatergic neurons.^12^ Previous findings suggested that drugs which directly or indirectly increased levels of glutamate, or that acted on postsynaptic sites to enhance glutamate receptor signaling, may produce rapid antidepressant‐like effects.^8^, ^27^ We, therefore, investigated the role of TREK‐1 on hippocampal synaptogenesis following AAV‐mediated knockdown or overexpression and pharmacological inhibition of CUMS‐exposed mice. In agreement with previous studies,^28^, ^29^ CUMS‐exposed mice showed a reduced number of the dendritic spines in the dentate gyrus and CA3 pyramidal cell layer. In addition, treatment with TREK‐1 inhibitors spadin and SID1900 counteracted CUMS‐induced reduction of spine density in the dentate gyrus and CA3 area. Furthermore, when compared to control mice and TREK‐1‐inhibited animals, CUMS‐treated mice showed marked significant atrophy of the apical dendrites of neurons in the hippocampus (dentate gyrus and CA3 areas). In addition, chronic stress reduced the expression of markers of synaptogenesis, including synaptic proteins PSD95 or synapsin, which were reversed by TREK‐1 inhibition, as well as neuron‐specific knockdown of TREK‐1 in the hippocampus. The changes in morphology combined with the upregulation of synaptic markers in TREK‐1 knockdown and TREK‐1 inhibited mice, consistent with the observations of treatment with spadin,^19^ strongly suggested that the TREK‐1 channel may be a potential therapeutic target of depression, and TREK‐1 inhibitors, such as spadin and SID1900, may be potent up‐regulators of neuronal functions.

Our study further revealed the cellular mechanism underlying the function of TREK‐1 in regulating synaptic plasticity in neurons after stress. We also analyzed the role of TREK‐1 on the synaptic plasticity in the CA1 region. Chronic stress impairs memory and LTP in the CA1 region of the hippocampus.^30^, ^31^ In agreement with the findings reported in previous studies, the results of the present electrophysiological studies in the hippocampal slice CA1 region showed that stress severely reduced the magnitude of LTP and that neuron‐specific TREK‐1‐knockdown prevented the stress‐related effects. Moreover, although stress has a profound effect on LTP, it does not impair the frequency of mEPSC but instead decreases its amplitude, thereby indicating that postsynaptic rather than presynaptic mechanisms may underlie the antidepressant effect of TREK‐1 knockdown. Combined, our findings not only support a synaptogenesis hypothesis of depression but also are consistent with previous studies that showed that chronic stress impairs hippocampal‐dependent cognition.

Our study has several limitations. First, we restricted our studies to a specific model of stress‐induced depressive‐like behaviors in mice, which may not completely translate to major depressive disorders in humans. Therefore, it would be of interest to validate the results in other animal models, including those of social defeat or learned helplessness. In addition, in our study, we have focused on the hippocampus as the site for gene knockdown and overexpression as well as for morphological studies. Other regions of the brain are dysfunctional in depression and deserve further TREK‐1 activity related investigations. We have yet to understand the specific neuronal systems involved beyond our initial findings relating to glutamatergic function. Moreover, behavioral tests are different in their ability to satisfy each validity. For example, the FST provides high predictive validity, but little construct or face validity.^32^ Additionally, the SPT has much higher face validity and greater construct validity than the more abstract immobility response to the FST.^33^ Given the limitations and the factors influencing responses to behavioral tests, it would be necessary to jointly analyze data from a set of related behavioral tests and further investigate in different strains and species.

Nevertheless, in the present study, we demonstrated that the TREK‐1 channel in the hippocampus is essential for depressive‐like behaviors in response to the CUMS‐induced anhedonia model of depression. Furthermore, the study demonstrated the modulatory effect of TREK‐1 on dendrite morphology, dendrite spine density, and synaptic plasticity in the hippocampus. We, therefore, proposed that TREK‐1 is involved in the pathogenesis of depression by regulating synaptogenesis. Combined, our findings provide an insight into the pathological mechanism of depression and indicate a potential novel target for improved treatment of depression.

## CONFLICT OF INTEREST

The authors declare no conflict of interest.

## Supporting information

App S1

App S2
