# Supplementary material for: Genetic and pharmacological inhibition of two‐pore domain potassium channel TREK‐1 alters depression‐related behaviors and neuronal plasticity in the hippocampus in mice
Source: CNS Neurosci Ther. 2020 Aug 30;27(2):220–32. doi: 10.1111/cns.13450 (PMC7816204; doi:10.1111/cns.13450)
Supplement: Supplementary file 1 — App S1 [file CNS-27-220-s001.docx]

Supplementary information for

**Genetic and pharmacological inhibition of two-pore domain potassium channel TREK-1 alters depression-related behaviors and neuronal plasticity in the hippocampus in mice**

Fangfang Wu*****, Hongbing Sun, Weigang Gong, Xiaoli Li, Zhaohui Pan, Han Shan, Zhijun Zhang*****

***Corresponding author:**

Zhijun Zhang, MD, Ph.D., Department of Neurology, Affiliated ZhongDa Hospital, School of Medicine, Southeast University, No. 87 Dingjiaqiao Road, Nanjing, Jiangsu, China, 210009; Tel: +86 25 83262241; E-mail: janemengzhang@vip.163.com

Fangfang Wu, Ph.D., Department of Neurology, Affiliated ZhongDa Hospital, School of Medicine, Southeast University, No. 87 Dingjiaqiao Road, Nanjing, Jiangsu, China, 210009; Tel: +86 25 83262241; E-mail: fangfang.wu@seu.edu.cn

**This file includes:**

Supplementary Methods

Figs. S1 to S5

Tables S1

References for SI reference citations

**SUPPLEMENTARY METHODS**

**Microinjection of TREK-1 shRNA and overexpression adeno-associated virus**

Using a more efficient adeno-associated virus (AAV) vector construct (AAV 2/8 serotype)[^1^](#_ENREF_1)^,^[^2^](#_ENREF_2), which uses the neuron-specific synapsin-1 (SYN) promoter to drive TREK-1 shRNA or TREK-1 over-expression, and includes a Woodchuck hepatitis virus post-transcriptional regulatory element (WPRE) enhancer element, we have been able to sufficiently genetically manipulate TREK-1 in neurons. The target region of shRNA TREK-1 in mouse was 5’-GCGTGGAGATCTACGACAAGT-3’. The full-length TREK-1 (NM_001159850) was synthesized to generate an overexpression vector, which was purchased from OBiO Technology Co., Ltd. (Shanghai, China).

Five-week-old male C57BL/6J mice were weighed before experiments and randomly assigned to different groups. Mice were anesthetized with sodium pentobarbital (45 mg/kg, i.p.). As an analgesic, carprofen (5 mg/kg s.c.) was administered before and every 24 h following surgery for 48 h. For intra-hippocampal microinjections of neuron-specific AAV, mice were placed in a stereotaxic apparatus. Animals that showed signs of distress before the stress program were removed from the experiment and euthanized. In all experiments, mice were only included for analysis when postmortem histological evidence showed an appropriately targeted microinjection, as visualized by the expression of the red-colored fluorescent protein mCherry.

**Microscopy and image analysis**

Mice were transcardially perfused with freshly prepared 4% paraformaldehyde. Then, brains were cut into 30-μm coronal slices using a cryostat and further processed for immunofluorescence staining of mCherry. Prolong gold anti-fade reagent containing DAPI (Southern Biotech, Birmingham, AL, USA, 0100-20) was applied for visualization of nuclei. Immunofluorescence images were captured using a microscope (OLYMPUS, Tokyo, Japan, DP73).

**Sucrose preference test (SPT)**

The sucrose preference test was carried out as previously described [^3^](#_ENREF_3). This test was conducted in the following in three phases: phase 1 habituation, phase 2 sucrose preference baseline, and phase 3 sucrose preference testing. In phase 1, 1% w/v sucrose solution was given to the mice for 3 days to habituate mice to the solution. In phase 2, each mouse was transferred to a single cage and was water deprived for 12 h, then exposed to both tap water and a sucrose solution for 12 h to obtain the sucrose preference baseline. Sucrose preference was then tested via a two-bottle choice test using standard bottles, one filled with tap water, the other with 1% sucrose solution, and was given to mice at 12 h after water deprivation for a total of 12 h (phase 3). The positions of the bottles were counterbalanced across the study to prevent the possible effect of side-preference in drinking behavior. The intake of tap water and sucrose solution was quantified by subtracting the final weight of bottles after 12 h exposure from their initial weight. The preference to consume the sucrose solution was calculated as a percentage of preference = [(sucrose intake/total intake) × 100]. Tests were performed by an individual who was blinded to the animal’s treatment status.

**Open field test (OFT)**

At 24 h after the sucrose preference test, mice were evaluated for spontaneous exploratory activity. In brief, individual mice were placed in the center of a large square box (50 × 50 × 50 cm) with a floor divided into 16 equal areas. Animals were permitted unrestricted exploration of the arena for a 5-min session and movements were digitally tracked using the ANY‐maze Video Tracking System (Stoelting Co., Wood Dale, IL, USA). The locomotor activity was analyzed afterwards. The time spent moving in the central zone and the distance moved (in cm) was calculated for each mouse using the ANY‐maze behavioral analysis software (version 4.3; Stoelting Co.).

**Forced swim test (FST)**

At 24 h after the open field test, the forced swim test was carried out as described previously with slight modifications [^3^](#_ENREF_3). In brief, individual mice were placed into an open cylinder container (diameter: 15 cm; height: 25 cm) filled with 15 cm water that was maintained at 22 ± 1 °C for 6 min. After vigorous activity during the first 2 min, mice acquired an immobile posture, which was characterized by motionless floating in an upright position with only minor movements to keep their heads above water. The duration of immobility was recorded during the last 4 min of the 6 min test by ForcedSwimScan™ (Clever Sys Inc., Reston, VA, USA). The immobility observed in this test was considered to reflect a state of despair. Tests were performed by an individual who was blinded to the animal’s treatment status.

**Slice electrophysiology**

**Brain slice preparation.** One day following the final exposure to stress and behavior tests, mice were sacrificed via deep isoflurane anesthesia and decapitated. Brain slices containing the hippocampus were prepared as previously described [^4^](#_ENREF_4). In brief, brains were rapidly removed, and 300 μm coronal slices were cut using a vibratome (Leica Biosystems, Buffalo Grove, IL, USA) in ice-cold Ringer solution. Slices were immediately placed in an interface chamber containing normal artificial cerebrospinal fluid (ACSF, in mmol/L: 124 NaCl, 3 KCl, 2 CaCl_2_, 26 NaHCO3, 1 MgSO4, 1.25 NaH_2_PO_4_, 10 glucose, bubbled with a 95% O_2_ – 5% CO_2_ mixture, pH 7.3) maintained at 30 ℃, and allowed to recover for at least 1.5 h. Subsequently, slices were placed in a holding chamber and continuously perfused at a rate of 1.5 mL min^-1^ with normal ACSF maintained at 30 ℃.

**Field potential recording.** Bipolar stimulating electrode were placed in the Schaffer collaterals to evoke a constant stimulation. The evoked field excitatory postsynaptic potentials (fEPSPs) were recorded in the stratum radiatum layer of CA1 region with a glass micropipette (3–5 MΩ resistance filled with 3 M NaCl). The fEPSPs were evoked by stimuli at 0.033 Hz (4–5 V, 20 μs). After recording of a stable baseline for at least 20 min, long-term potentiation (LTP) was induced by three trains of high-frequency stimulation (HFS) conditioning pulses (100 Hz for 1 s, 30 s interval). The fEPSPs were monitored for 60 min. The intensity of the stimulation was adjusted to produce an fEPSP with an amplitude of 30–40% of the maximum response. Slopes of fEPSPs were normalized to the average of the slopes of the fEPSPs that were acquired during baseline. All data were calculated as the average of the last 15 min out of 60 min of recordings.

**Whole-cell patch-clamp recordings.** Cortical slices were transferred one at a time to a submersion recording chamber and allowed to equilibrate for 10 min prior to recordings. Whole-cell voltage-clamp recordings of CA1 pyramidal cells were performed with a MultiClamp 700B amplifier (Molecular Devices, Sunnyvale, CA, USA) as previously described [^4^](#_ENREF_4). Slices were recorded with patch electrodes (3–6 MΩ resistance) filled with a solution containing (in mM): 122.5 Cs-gluconate, 17.5 CsCl, 0.2 EGTA, 10.0 HEPES, 1.0 MgCl_2_, 4.0 Mg-ATP, 0.3 Na-GTP, and 5.0 QX314, pH 7.2 (280–300 mOsm). The miniature excitatory postsynaptic current (mEPSC) was isolated by including bicuculline (20 μM) and tetrodotoxin (1 μM) in the bath solution. All recordings were performed at room temperature at a holding potential of -70 mV. Data was filtered at 2 kHz and sampled at 10 kHz using Digidata 1322A digitizer (Molecular Devices, Sunnyvale, CA, USA), then acquired using pClamp 10 software. Data were analyzed by the Mini Analysis Program (Synaptosoft, Decatur, GA, USA) with an amplitude threshold of 5 pA for mEPSC analysis.

**Western blot analysis**

Hippocampal tissues were collected and extracted in RIPA lysis buffer (Beyotime, Shanghai, China, P0013B). Protein concentrations were measured using the Pierce BCA Protein Assay Kit (Pierce Biotechnology, Rockford, IL, USA). Proteins were separated by sodium dodecyl sulfate-polyacrylamide gel (10% or 12%) electrophoresis and electrophoretically transferred onto polyvinylidene fluoride membranes. Membranes were blocked with 5% non-fat dry milk in Tris-buffered saline with Tween-20 (TBST), incubated overnight with primary antibodies (see below) overnight at 4°C, and incubated with horseradish peroxidase (HRP)-conjugated goat anti-mouse/rabbit IgG secondary antibodies (1:2000, Cell Signaling, 7076P2/7074P2) and donkey anti-goat IgG secondary antibody (1:5000, Abcam, ab6885). The primary antibodies used were as follows: TREK-1 (1:200, Alomone Labs, Jerusalem, Israel, APC-047), synaptophysin (1:500, Abcam, Cambridge, MA, USA, ab8049), synapsin-1 (1:250, Santa Cruz, Santa Cruz, CA, USA, sc-8295), PSD95 (1:1000, Abcam, ab76115) and β-actin (1:1000, CMCTAG, AT0001). Membranes were visualized and imaged using Image Quant LAS4000 mini (GE Healthcare UK Ltd., Buckinghamshire, UK). Quantification of individual protein bands was performed by densitometry using ImageJ software (NIH, http://rsbweb.nih.gov/ij/). Moreover, the specificity of anti-TREK-1 antibody (Alomone, APC-047) was determined according to the manufacturer’s protocol. The results of antibody alone versus antibody with negative control antigen were provided in the Supplementary Figure 1.

**Real-time polymerase chain reaction (PCR)**

Real-time PCR was performed according to our previous studies in an Applied Biosystems Real-Time PCR System [^2^](#_ENREF_2). Total RNA was extracted using TRIzol reagent (Invitrogen, Carlsbad, CA, USA, 15596026). The mRNAs were reverse transcribed using a HiScript Q RT SuperMix for qPCR Kit (Vazyme, Nanjing, China, R123-01) and quantified using SYBR Green Real-time PCR Master Mix (Vazyme, R141-02). GAPDH was used as an internal control. The primers used to amplify the mRNA transcripts were synthesized by Invitrogen. The sequences of the primers are listed in Supplementary Table 1.

**Supplementary Figures**

**
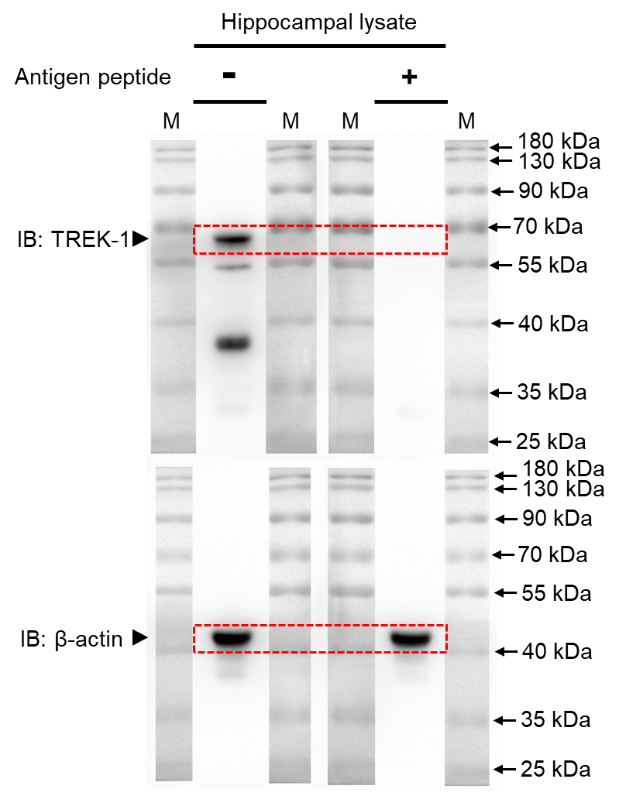
**

**Figure S1. Expression of TREK-1 two-pore domain potassium channels in hippocampal tissue.** Western blot analysis of mouse hippocampal lysates using Anti-TREK-1 Antibody (#APC-047), (left upper panel). In sample probed with the antibody preincubated with the negative control antigen (right upper panel), no signal is observed for TREK-1, showing that the antibody specifically detects TREK-1. Lower panel, immunoblot of β-actin. M, marker.

**Figure S2. Expression of two-pore domain potassium channels in hippocampus after microinjection of neuron-specific TREK-1 shRNA AAV virus.** Specificity of the TREK-1 knockdown was assessed by qPCR in hippocampal tissue at 4 weeks after AAV microinjection. n = 6 animals per group. ***p* < 0.01 vs. AAV-Con group using Student’s *t*-test. Data are shown as mean ± s.e.m.

**Figure S3.** **Effect of neuron-specific knockdown of TREK-1 in hippocampus on locomotor activity.** Specific knockdown of TREK-1 expression in neurons exerted no significant effect on the total distance in the OFT. n = 13 animals per group. Numbers in bars, numbers of mice. Data are shown as mean ± s.e.m. OFT, open-field test.

**Figure S4. Effect of neuron-specific overexpression of TREK-1 in hippocampus on locomotor activity.** Specific overexpression of TREK-1 expression in neurons exerted no significant effect on the total distance in the OFT. n = 10 animals per group. Numbers in bars, numbers of mice. Data are shown as mean ± s.e.m. OFT, open-field test.

**Figure S5. Effect of TREK-1 inhibitors on locomotor activity.** TREK-1 inhibitors exerted no significant effect on the total distance in the OFT. n = 10, 10, 11, 13, 13, and 13 animals in the control + saline, control + spadin, control + SID1900, CUMS + saline, CUMS + spadin, and CUMS + SID1900 groups, respectively. Numbers in bars, numbers of mice. Data are shown as mean ± s.e.m. OFT, open-field test.

**Table S1. The primer sequences used for qPCR analysis.**

| List of oligonucleotide sequences | 5’ > 3’ |
| --- | --- |
| GAPDH(mouse)-F | AGGTCGGTGTGAACGGATTTG |
| GAPDH(mouse)-R | TGTAGACCATGTAGTTGAGGTCA |
| Kcnk1 (mouse)-F | AGCAAAGTGAGCCTTTTGTGG |
| Kcnk1 (mouse)-R | GTGCATACCTCATTTGCCCA |
| Kcnk2 (mouse)-F | GAGAGACCCGGCTATACTGC |
| Kcnk2 (mouse)-R | GCCTCGGTTTGGAGTTCTGA |
| Kcnk3 (mouse)-F | CTACCTGCAACCCAGTGGAG |
| Kcnk3 (mouse)-R | CCATAGCCCCACACATACCC |
| Kcnk4 (mouse)-F | ACAAGACAGCATTGGGGAGC |
| Kcnk4 (mouse)-R | GGTCTCGGCCATGATCCATT |
| Kcnk5 (mouse)-F | GTGGGTCCTGAGTTTCACCC |
| Kcnk5 (mouse)-R | GATCACGTTCTCACAGGCCA |
| Kcnk6 (mouse)-F | CTATGGCTACACGACCCCAC |
| Kcnk6 (mouse)-R | TAGCATGGTGATAGGCACGC |
| Kcnk7 (mouse)-F | CGATACCTGCTCCTGCTTATGG |
| Kcnk7 (mouse)-R | ATCCCAGTTGCTTGTCTCTGA |
| Kcnk9 (mouse)-F | CATTTCTTTCCGGCCTTTCCAG |
| Kcnk9 (mouse)-R | CTAGGAAAGCCACCCCAGAG |
| Kcnk10 (mouse)-F | TCTTCCTCCTTTGGTGGCCG |
| Kcnk10 (mouse)-R | CCACCAGTGACGAGGTAGAC |
| Kcnk12 (mouse)-F | GTGTTCTCTGCGCTCGAAAG |
| Kcnk12 (mouse)-R | CCCACGAAGTAGAAGGCTCC |
| Kcnk13 (mouse)-F | TGCGTTTGGGAAGCGGTC |
| Kcnk13 (mouse)-R | CTGTGATGGCTGGGATACGG |
| Kcnk15 (mouse)-F | GCGAGCGTCTAAACACACTG |
| Kcnk15 (mouse)-R | CCGAAGCCTATGGTGGTGAG |
| Kcnk16 (mouse)-F | AAGGCAGGAAAGGTTCGTCC |
| Kcnk16 (mouse)-R | TCCTGGGTGGCAGTAAGACT |

**SUPPLEMENTARY REFERENCES**

1. Li WG, Liu MG, Deng S, et al. ASIC1a regulates insular long-term depression and is required for the extinction of conditioned taste aversion. *Nature communications.* 2016;7:13770.

2. Wu F, Han B, Wu S, et al. Circular RNA TLK1 Aggravates Neuronal Injury and Neurological Deficits after Ischemic Stroke via miR-335-3p/TIPARP. *J Neurosci.* 2019;39(37):7369-7393.

3. Du RH, Wu FF, Lu M, et al. Uncoupling protein 2 modulation of the NLRP3 inflammasome in astrocytes and its implications in depression. *Redox biology.* 2016;9:178-187.

4. Li MX, Zheng HL, Luo Y, et al. Gene deficiency and pharmacological inhibition of caspase-1 confers resilience to chronic social defeat stress via regulating the stability of surface AMPARs. *Mol Psychiatry.* 2018;23(3):556-568.
