# Supplementary material for: Genetic and pharmacological inhibition of two‐pore domain potassium channel TREK‐1 alters depression‐related behaviors and neuronal plasticity in the hippocampus in mice
Source: CNS Neurosci Ther. 2020 Aug 30;27(2):220–32. doi: 10.1111/cns.13450 (PMC7816204; doi:10.1111/cns.13450)
Supplement: Supplementary file 2 — App S2 [file CNS-27-220-s002.pdf]

Supplementary information for

Genetic and pharmacological inhibition of two-pore domain potassium channel TREK-1 alters depression-related behaviors and neuronal plasticity in the hippocampus in mice

Fangfang Wu*, Hongbing Sun, Weigang Gong, Xiaoli Li, Zhaohui Pan, Han Shan , Zhijun Zhang*

***Corresponding author:**

Zhijun Zhang, MD, Ph.D., Department of Neurology, Affiliated ZhongDa Hospital, School of Medicine, Southeast University, No. 87 Dingjiaqiao Road, Nanjing, Jiangsu, China, 210009; Tel: +86 25 83262241; E-mail: janemengzhang@vip.163.com

Fangfang Wu, Ph.D., Department of Neurology, Affiliated ZhongDa Hospital, School of Medicine, Southeast University, No. 87 Dingjiaqiao Road, Nanjing, Jiangsu, China, 210009; Tel: +86 25 83262241; E-mail: fangfang.wu@seu.edu.cn

This file includes:

Original gel/blot images of each cropped gel/blot appearing in the manuscript

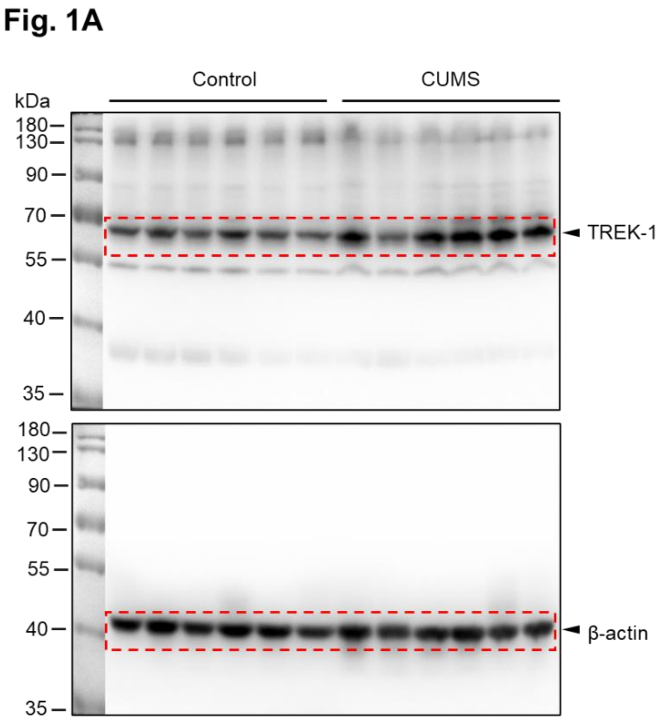

Figure 1-1. Full unedited gel/blot for Figure 1A.

Fig. 1F

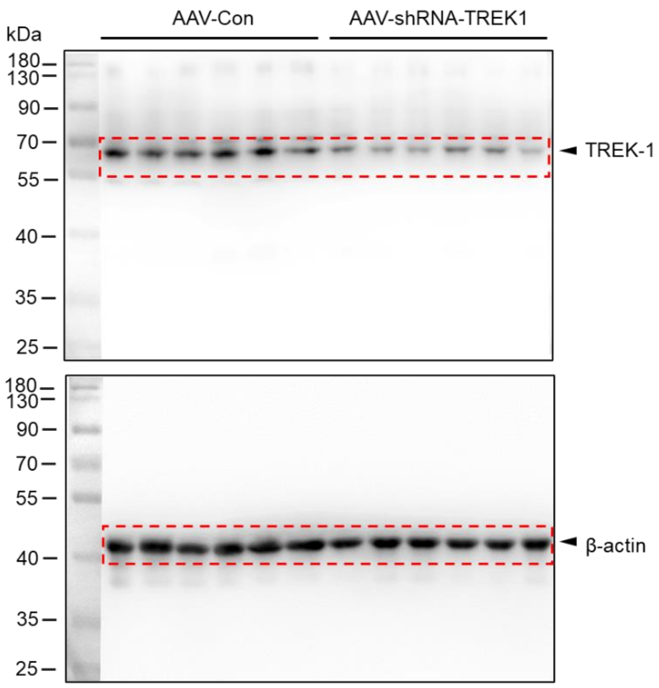

Figure 1-2. Full unedited gel/blot for Figure 1F.

Fig. 2D

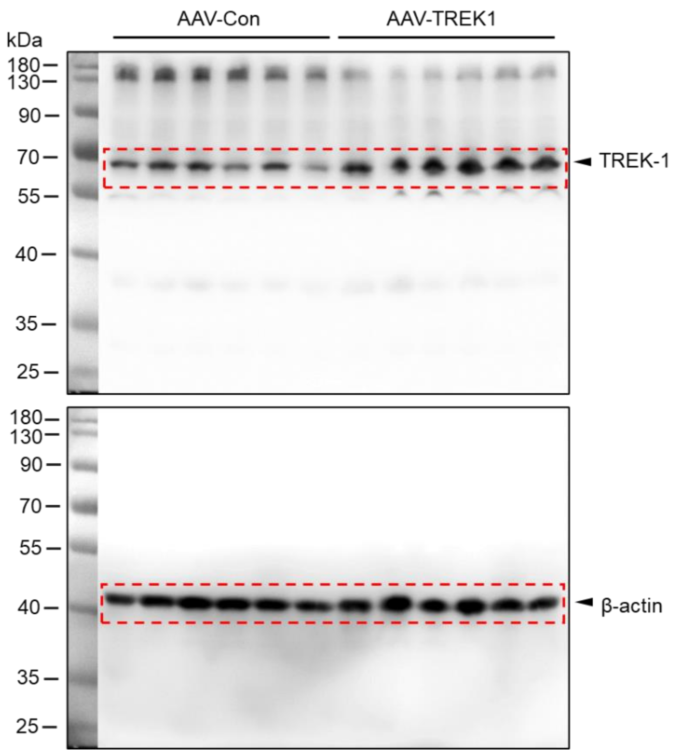

Figure 2-1. Full unedited gel/blot for Figure 2D.

Fig. 3F

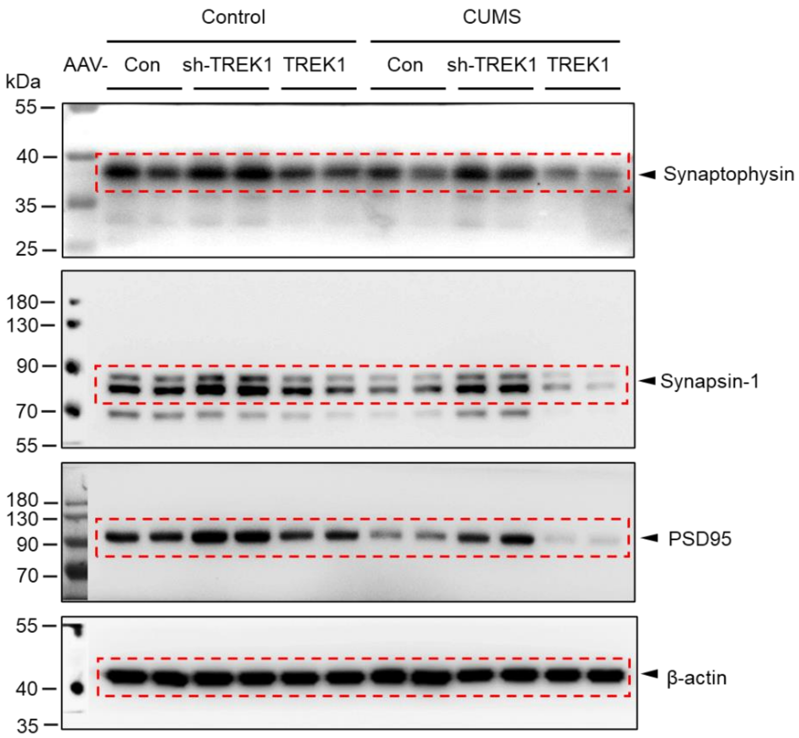

Figure 3-1. Full unedited gel/blot for Figure 3F.

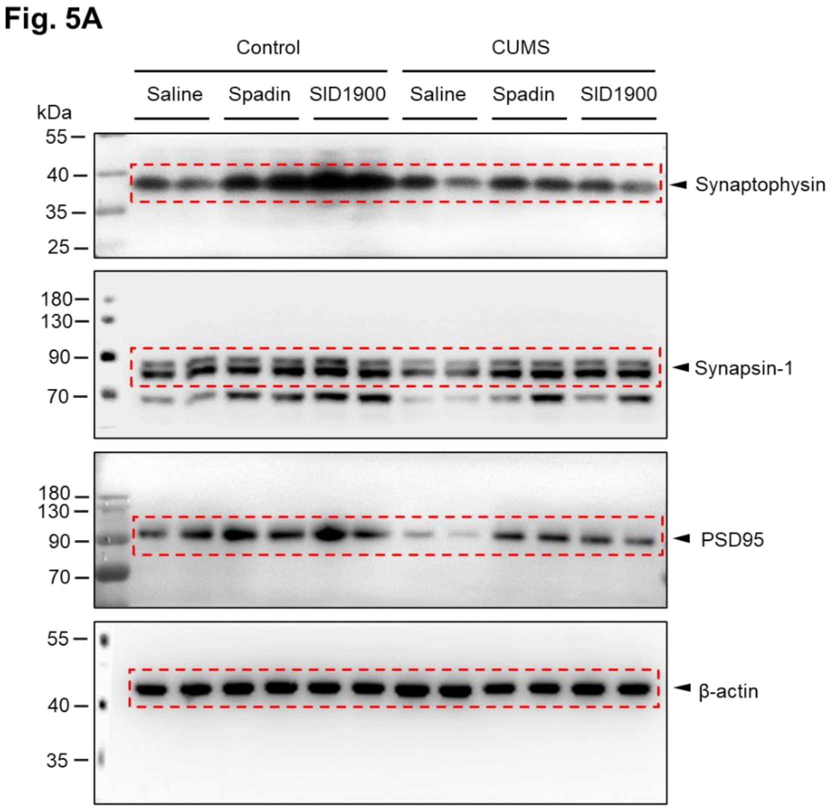

Figure 5-1. Full unedited gel/blot for Figure 5A.
